# Histopathology of thymectomy specimens from the MGTX-trial: Entropy analysis as strategy to quantify spatial heterogeneity of lymphoid follicle and fat distribution

**DOI:** 10.1371/journal.pone.0197435

**Published:** 2018-06-13

**Authors:** Cleo-Aron Weis, Inmaculada B. Aban, Garry Cutter, Henry J. Kaminski, Christoph Scharff, Benedict W. Grießmann, Maria Deligianni, Klaus Kayser, Gil I. Wolfe, Philipp Ströbel, Alexander Marx

**Affiliations:** 1 Institute of Pathology, University Medical Centre Mannheim, University of Heidelberg, Mannheim, Germany; 2 Department of Biostatistics, University of Alabama at Birmingham, Birmingham, AL, United States of America; 3 Department of Neurology, George Washington University Medical Center, Washington, DC, United States of America; 4 Institute of Pathology, Charité, Berlin, Germany; 5 Department of Neurology, University at Buffalo Jacobs School of Medicine and Biomedical Sciences, Buffalo, NY, United States of America; 6 Institute of Pathology, University Medical Center Göttingen, University of Göttingen, Göttingen, Germany; University of Sydney, AUSTRALIA

## Abstract

The thymectomy specimens from the “thymectomy trial in non-thymomatous myasthenia gravis patients receiving prednisone therapy” (MGTX) underwent rigid and comprehensive work-up, which permits analysis of the spatial distribution of histological and immunohistological features. This analysis revealed strong intra- and inter-case variability. While many histological features (e.g. median percent fat content among different specimens) can easily be correlated with clinical parameters, intra-case spatial variability of histological features has yet defied quantification and statistical evaluation. To overcome this gap in digital pathology, we here propose intra-case entropy of measured histological features in all available slides of a given thymectomy specimen as a quantitative marker of spatial histological heterogeneity. Calculation of entropy led to one value per specimen and histological feature. Through these ‘entropy values’ the so far neglected degree of spatial histological heterogeneity could be fed into statistical analyses, extending the scope of clinico-pathological correlations.

## Introduction and objectives

**Fig 1:**
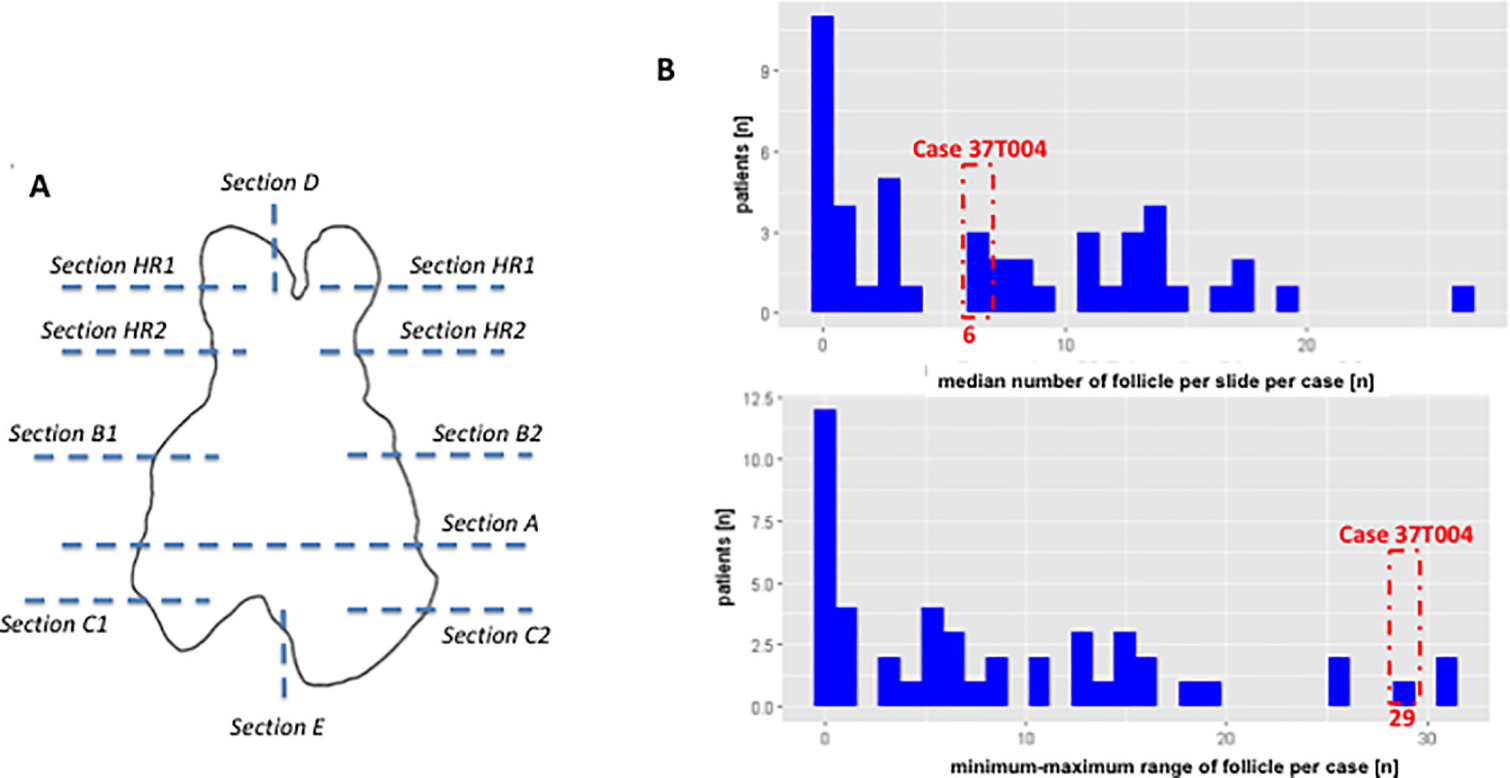
Specimen work-up and heterogeneous spatial distribution of the number of follicles on one slide A) Work-up scheme for the specimens in the MGTX trial [1, 3]. Thymuses were sub-divided into regions that underwent predefined evaluation (e.g. complete work-up of the central A-region, partial work-up of other regions) B) Distribution of the median number of lymphoid follicles in CD23-stained sections per case and the associated minimum-maximum-value-range across all available slides per case. Case 37T004 from the MGTX-trial is highlighted as an example showing high in-case variation.

The “thymectomy trial in non-thymomatous myasthenia gravis patients receiving prednisone therapy” (MGTX) [1] showed that extended trans-sternal thymectomy in combination with prednisone was significantly more beneficial than prednisone alone in terms of myasthenia gravis (MG) clinical status and corticosteroid requirements. The study protocol included a rigid method for handling and inspection of thymectomy specimens (Fig 1A) [2]. This standardization has allowed i) the calculation of overall, mean or median values of various immunohistological features (e.g. the mean number of CD23(+) lymphoid follicles per slide per thymectomy specimen), and ii) analysis of the spatial distributions of immunohistological features across different anatomical regions of individual thymectomy specimens and across specimens of the thymectomized cohort.

The objective of this analysis is to correlate quantitative histological findings (including their heterogeneity as a new morphological ‘dimension’) with clinical outcome parameters to eventually identify at the time of surgery pathological features with prognostic value in terms of MG outcome. Furthermore, it is hoped that recommendations for an appropriate and economical evaluation of thymectomy specimens will result from the analysis.

## Material

### 1.1 Thymectomy specimens

Thymectomy specimens were retrieved at different clinical centers and processed according to the MGTX protocol, including removal of all mediastinal fat from the thymus proper [1–3]. This protocol requested local pathologists to retrieve numerous tissue blocks of defined size from strictly defined bilateral regions of the formalin fixed thymectomy specimens (see below for details) [3, 4]. This rigid, spatially standardized sampling scheme enabled us to comparatively analyse spatial tissue heterogeneity among the resection specimens from different patients [3, 4]. From the 66 thymectomy specimens that were recruited for the MGTX trial, 11 dropped out because i) local pathologists disregarded the sampling protocol; ii) the diagnosis was incorrect (thymoma instead of non-neoplastic thymus); or iii) patients withdrew their consent to the scientific evaluation of their resected thymuses. Therefore, 55 cases were included in the current study. Each thymus was subdivided into several predefined sub-regions (Fig 1A). The central horizontal slice of each formalin-fixed specimen (plane / region A) underwent complete work-up, while representative single sections of at least 1 cm^2^ were obtained from defined regions of the right and left thymic lobe above and below the central plane (Fig 1A). All blocks with formalin-fixed, paraffin-embedded tissue from the 55 cases were finally submitted to the Institute of Pathology, University Medical Centre Mannheim, Germany.

### 1.2 Evaluation of histological parameters

Of the 55 thymectomy specimens, all obtained tissue blocks (11±5 blocks per specimen) underwent a pre-defined standard diagnostic protocol: First, all sections were hematoxylin-eosin (HE)-stained and evaluated for percentage of fat tissue on the slide; percentage of intra-thymic fat tissue; grading of cortical atrophy; grading of follicular hypertrophy; number of follicles, and proportion of cortical and medullary areas (Table 1). Second, slides containing thymic parenchyma were immunohisto-chemically stained for CD23 (expressed by follicular dendritic cells) using a routine immunoperoxidase technique [5, 6]. Numbers and morphology of lymphoid follicles were ‘manually’ assessed on digitalized sections (Table 1).

**Table 1:** Overview of gathered histomorphological data. For most of the slides of the 55 thymectomy specimens 16 histomorphological parameters were collected using different means of measurement as indicated in column. Region “A” designates the completely processed central horizontal tissue plane of a given thymus as shown in Fig 1A. HE, hematoxylin and eosin.

Variable	Mode of acquistion	Regions analyzed	Staining	Value range	Measurement scale	Basic summary statistics
Narrative description of follicle morphology	Visual inspection	all	HE, CD23		nominal	
Grading atrophy	Visual inspection	all	HE	grades 0–4	ordinal	Grade 2 (modus)
Grading follicular atrophy	Visual inspection	all	HE, CD23	grades 0–4	ordinal	Grade 0 for HE / Grade 1 for IHC (modus)
Grading overall fat content	Visual inspection and estimation	all	HE	0–100%	interval	66.6±24.7% (mean±std)
Grading intrathymic fat content	Visual inspection and estimation	all	HE	0–100%	interval	46.9 ± 21.3%(mean±std)
Area with B-cellular infiltrate	Visual inspection and estimation	all	CD20	0–100%	interval	31.2±16.9%(mean±std)
Number of follicle	Visual inspection and counting	all	HE, CD23	0–100	interval	1.2±2.5 follicles (mean±std) for HE / 7.1±6.7 follicles (mean±std) for IHC
Cortical area	Automatic image processing	all	HE	0–10.000pixel	interval	2.9±1.4 mm^2 (mean±std)
Medullary area	Automatic image processing	all	HE	0–10.000pixel	interval	13.1±6.9 mm^2 (mean±std)
Follicle area	Manual segmentation	A	HE, CD23, IgD	0–10.000pixel	interval	18964±14225 μm^2 (mean±std)
Germinal centre area	Manual segmentation	A	HE, CD23, IgD	0–10.000pixel	interval	7877.5±6466.3 μm^2 (mean±std)
Mantle zone area	Manual segmentation	A	HE, CD23, IgD	0–10.000pixel	interval	12510±8496.2 μm^2 (mean±std)
Marginal zone area	Manual segmentation	A	HE, CD23, IgD	0–10.000pixel	interval	27116.8±17851.9 μm^2 (mean±std)
Area thymic tissue	Manual segmentation	A	HE	0–10.000pixel	interval	8820.7±6609.9 mm^2 (mean±std)
Number of follicle with germinal centre	Visual inspection and counting	A	HE	0–100	interval	12.7±12.6 follicles (mean±std)
Number of follicle without germinal centre	Visual inspection and coutning	A	HE	0–100	interval	5.3±6.7 follicles (mean±std)

This process led to 16 histological variables (Table 1) per slide (e.g. percentage of fat tissue as interval scaled data (0–100%); grading of thymic follicular hyperplasia (grades 0–4) as ordinal scaled data) [4]. Nine of these parameters (e.g. the number of lymphoid follicles per slide) were derived on the basis of the visual inspection of histological slides by two pathologists (CW and AM), i.e by a technique that can realistically be integrated into a routine pathology workflow. For the semiautomatic quantification of 5 different types of areas (e.g. the area of lymphoid follicles) we applied manual segmentation on a subset of the slides. Finally, two parameters (e.g. cortical area) were assessed by fully automatic image processing in all available slides. On the basis of these data, statistical “location parameters” (mean, median, modus) were calculated in order to correlate quantitative histological features with clinical outcome parameters.

### 2.3 Data accessibility

The raw data we are dealing with in this work has been collected in the course of the MGTX trial and is centrally managed by the MGTX Data Coordinating Center [3, 4]. The data described and used in this work is available at heiData (http://dx.doi.org/10.11588/data/NWE2JJ).

### 2.4 Data management and gathering in R

As described above (Table 1) data were produced using very different approaches (e.g. visual inspection with estimation of cortical atrophy; grading or counting; semi-automatic image processing in Fiji (www.fiji.sc) and automatic image processing in MATLAB (MATLAB R2016a, Mathworks, Natick, MA, USA) and saved in spreadsheets (Microsoft Excel 2010, Microsoft Corporation, Redmond WA, USA). Furthermore, demographic and clinical data associated with each case were obtained from Dr. Cutter, University of Alabama at Birmingham and submitted in spreadsheets.

All these datasheets were gathered in R (www.r-project.org) and merged into two main databases: one database with values per slide and another database with summation values per case (on the basis of unique case IDs).

### 2.5 Statistical methods applied

All statistical analysis were performed in R (www.r-project.org) by built-in functions or separately loaded libraries [7]:

P-values were calculated accordingly after testing for normal distribution using the Shapiro-Wilk test [7, 8]. T-test and Wilcoxon-test were performed in case of normally distribution and non-normally distributed variables, respectively. P-values <0.05 were considered as significant.

Pearson-correlation were calculated to check for the linear correlation between two variables [7].

Linear modelling was performed with single or multiple independent variables to check for their correlation and prediction quality regarding the modelled endpoints [7, 8].

### 2.6 Evaluation of entropy as marker of spatial heterogeneity

Since statistical “location parameters” (e.g. means, medians) are not helpful to evaluate “intra-case variance”, i.e. spatial heterogeneity of a particular histological feature in a given case (as depicted for follicle counts in Fig 1B), we first quantified variance as an additional feature [9]. Since summation of the deviation values per feature (e.g. range of the follicle number or standard deviation of fat content) can describe the variance of one feature, but is not suitable to measure the compound spatial heterogeneity of variables with different measurement scales, we here propose a probability based model for the quantification of heterogeneity based on entropy measurement (in bit) per case [10]. This strategy has been successfully used in other scientific disciplines such as ecology [10] and digital image processing [11–14]. In these fields, entropy was defined in terms of information theory [15, 16] and has been used to quantify spatial heterogeneity.

**Fig 2:**
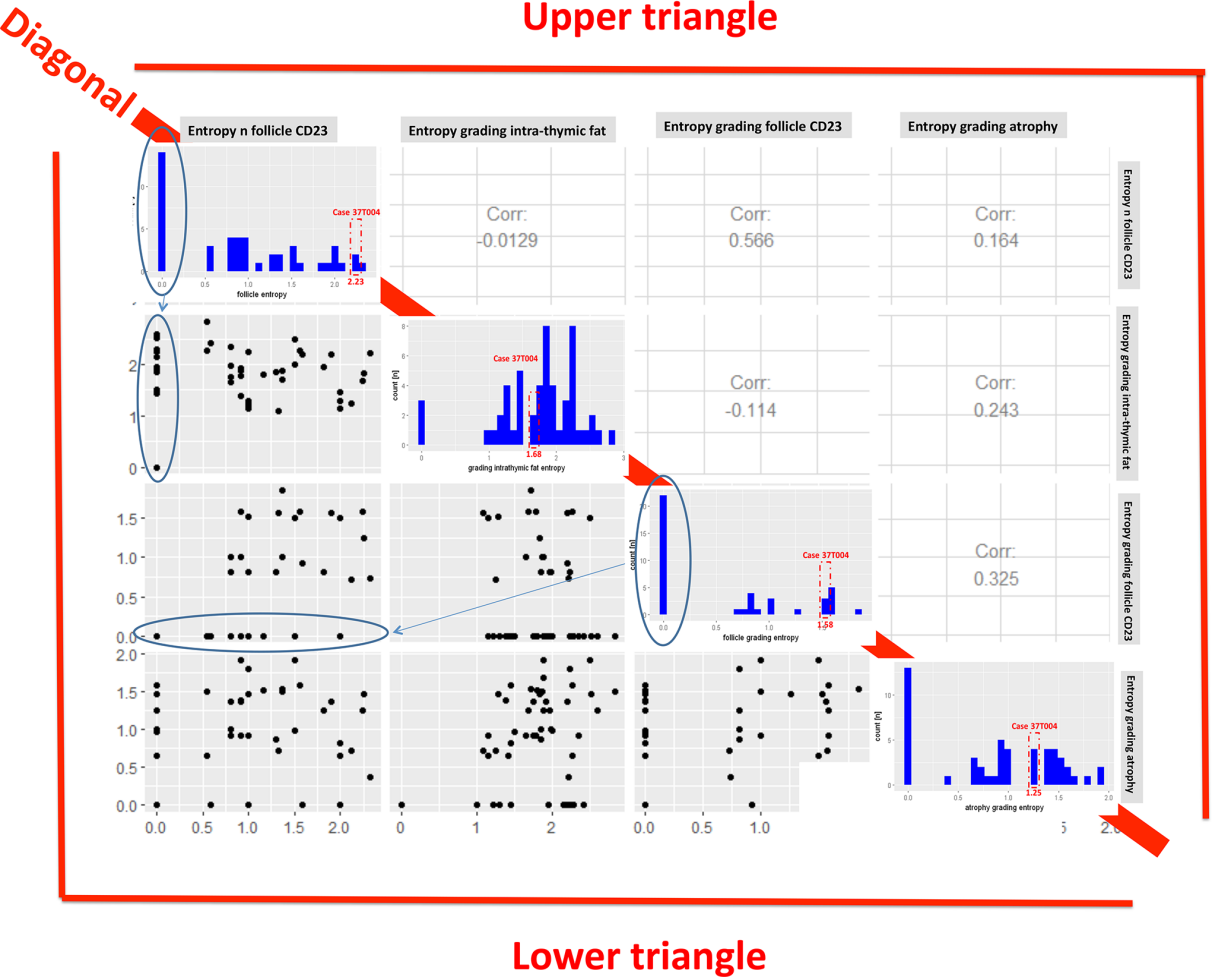
Variance of the entropy in the MGTX collective. Entropy was calculated for the number [n] of follicles and for the grading of lymphofollicular hyperplasia in CD23-stained sections [grades 0–4]; entropy calculations for the grading of intra-thymic fat content [%] and the grading of thymic atrophy [grades 0–4] were based on measurements based on HE-stained sections. Diagonal: The histogram per entropy is plotted with case 37T004 being highlighted. Lower triangle: Below the diagonal the variables are plotted against each other. Upper triangle: Above the diagonal the corresponding correlation coefficients are plotted.

This approach can be divided into several steps: i) For the sake of simplification, every region (measurement point) could have one pre-defined level per variable from a certain range (e.g. number of lymph follicles with levels 1–10 in Fig 2A; atrophy grading with levels 0 to 4 in Fig 2B). The rationale behind choosing and defining the numbers of levels and the thresholds were to not over estimate (e.g. in the case of estimated fat-content 5% and 8% are not significant different) and not to under estimate heterogeneity (e.g. two levels for a fat-content ranging from 0–100%).This is analogous to digital image processing [12], where entropy calculation of grayscale images with intensities between 0 and 256 (for a 8bit image) is achieved through reduction of the 256 intensities to 16 intensity levels. ii) For these levels the relative probability can be calculated (compare Fig 2). In case of complete randomness the probability of every level (*p*_*i*_) should converge to one divided by the number of measurement points / region (*m*^*-1*^). Then, according to Shannon [10, 16, 17], entropy *H* can then be calculated as $H=-\sum_{i=1}^{m}p_{i}\;{log}_{2}(p_{i})$ with *m* as the number of measurement points and p_i_ as the relative probability of the level of the measurement point *i*.

iii) Entropy can be defined per variable (e.g. number of lymphoid follicles, atrophy grade) and case (depending on the number of measurement points), which results in a maximum possible entropy per variable as a function of the number of measurement points and the probability of the examined feature. Accordingly, when 6 slides (measurement points) per case were studied, the respective entropy is $H_{max}=-\sum_{i=1}^{6}\frac{1}{5}\;{log}_{2}\left(\frac{1}{5}\right)=2.786$ for the atrophy grading that was subdivided into 5 levels (grades 0–4) and $H_{max}=-\sum_{i=1}^{6}\frac{1}{10}\;{log}_{2}\left(\frac{1}{10}\right)=1.993$ for the number of lymphoid follicles, since their absolute numbers (range = 0–50) were subdivided into 10 levels (levels 1–10). If there are identical values in all regions, i.e. if there is a completely random distribution, the entropy $H_{max}=-\sum_{i=1}^{6}1\;{log}_{2}(1)=0$ (compare Fig 2).

## Results

### 3.1 Thymic histomorphological heterogeneity across the cohort of MGTX-patients

Based on HE-stained (for fat) or CD23-stained (for follicles) sections, entropy per case was calculated for four histological features: The number of follicles; the grade of lymphofollicular hyperplasia (based on follicles per low power field)[4]; the grade of atrophy and the estimated percentage of intra-thymic fat) (Fig 2). The entropy values showed a remarkable variability for all features across the cases (compare the histograms on the diagonal in Fig 2). Furthermore, the entropy values of each single feature showed no significant correlation with any one of the other three features. Therefore, the entropies of these four histological features of the thymectomy specimens appeared independent of each other and usable as independent variables for further statistical analyses.

### 3.2 Comparing histological parameters and their entropy

Entropy of histological parameters (e.g. the percentage of intrathymic fat, the number of CD23-positive lymphoid follicles etc.) was calculated in order to generate new variables to be used for further statistics and modeling [9]: Here, we found no evidence of a correlation between the entropy of the grading of the intra-thymic fat content and the patient age (Pearson correlation -0.12). This finding is in contrast to the percentage of intra-thymic fat itself that needs normalization to age because of thymic involution (Pearson correlation 0.47 and p <0.001 for the correlation of intrathymic fat and the patient age)[3, 18]. Interestingly, we found a marginally non-significant correlation between the percentage of intrathymic fat and the BMI as surrogate of the body weight (Pearson correlation 0.44, p = 0.051). In turn, for the entropy of the intrathymic fat there is no correlation to the BMI.

Furthermore, we found that that the entropy of the number of CD23-positive follicles was not significantly different between patient groups stratified according to basic patient parameter like gender (mean entropy in females 0.52±0.66, mean entropy in males 0.47±0.60, p = 0.786) and age (Pearson correlation across an age range of 18–68 years was -0.23, p = 0.319).

This could indicate that entropy of these particular features is an independent variable, which does not need normalization to patient age and gender.

### 3.3 Checking the independence of entropy

One possible limitation of the entropy approach is its relation to the number of measurement points per case: If there are only few points (e.g. due to a small specimen or to limited sampling), the maximal possible entropy of that case is relative low (e.g. for 3 measurement points with 10 possible levels (p_i_ = 0.1) H_max_ = 0.99). This low value of entropy could lead to the erroneous conclusion that the investigated variable shows a high degree of order. To test for this possibility, we correlated the calculated entropy values with the number of underlying measurement points. Indeed, there was no significant correlation between the entropy values for the number of follicles and the number of measurement points, i.e. slides per case (Pearson-correlation 0.07, p = 0.652). We interpret this finding as an indication of having enough sample points per case to avoid over-estimation of the entropy of the number of follicles.

### 3.4 Correlation of histomorphological heterogeneity with clinical parameters

**Fig 3:**
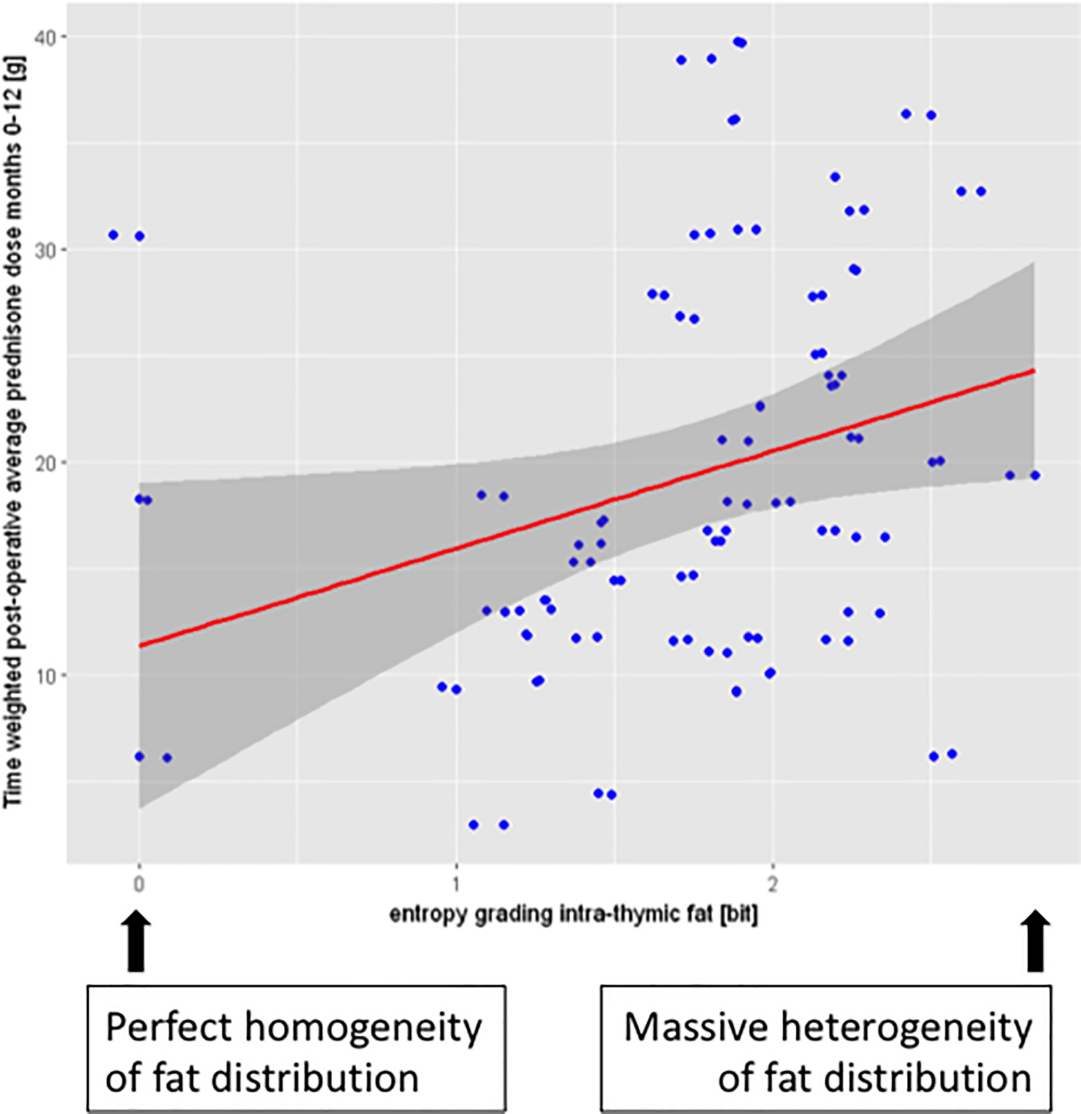
Correlation between the entropy of intra-thymic fat and post-operative absolute prednisone-dose. On linear modelling, a significant correlation (p = 0.03) between the entropy of intra-thymic fat per case and the post-operative absolute prednisone dose (area under the dose-time curve for prednisone months 0–12) could be shown. To visualize the correlation the values per case are plotted against each other with the calculated regression line.

We next analyzed the correlation between the entropy of the four histological parameters and several clinical parameters by explorative modeling (compare Table 1) [19, 20]. Thereby, the main idea was to include many variables in the linear model and to subsequently further focus on the ones with a significant contribution: The following clinical outcome parameters were analyzed separately: prednisone exposure (area under the dose-time curve) from the time point of surgery to 12 months after surgery; MG-severity (measured by the QMG-score) at enrollment; MG-duration before surgery; a 3-point drop in the QMG score (which has been defined as significant improvement by neurologists [1, 21, 22]), and the achievement or not of minimal manifestation status between month 12 and month 36 after surgery. On this analysis, only the entropy of the grading of intra-thymic fat content showed a statistically significant contribution in a linear regression model for the absolute post operative prednisone dose (henceforth called post-operative prednisone-load) (Table 2). Subsequently, the factors with a significant contribution to the model were analyzed alone. Then, a significant positive correlation between the entropy of the grading of the intra-thymic fat content (as single independent variable) and the postoperative prednisone load could be confirmed (p-value for entropy grading intra-thymic fat 0.031). Fig 3 illustrates the trend to higher post-operative prednisone loads for cases with higher entropy. However, since the correlation analysis showed a small R-squared (R^2^ = 0.071), the predictive power of our model was low. In order to check, whether our model was suitable at all for this set of data, we performed cross validation by randomly dividing our patient collective of n = 55 into three sub-groups (folds) of similar size, of which two subgroups were selected to serve as training set for the model, while the remaining third subgroup served as test set. The mean squared error of the three folds was 82.9±11.2g (prednisone load) as compared to a mean squared error of 82.1g when the model was trained on all data. Thus, there seems to be no over fitting.

**Table 2:** Explorative modelling. To model the effect of the entropy on certain clinical parameters / endpoints, explorative modelling with logistic regression for categorical clinical endpoints (QMG-drop, MMS) and linear regression for clinical endpoints with linear scaling (QMG-score, MG-duration, pre- and postoperative prednisone load) were performed. Respectively, one clinical parameter is thereby modeled on basis of four entropy values (for the number of follicles in the CD23-staining, the follicle grading in the CD23-staining, the grading of the intratyhmic fat and the grading of the atrophy). The table shows the p-value (* indicates significance at the 5%-level) per variable in the adjusted model that contains all the other variables against the respective clinical endpoint.

	entropy [bit]					
end-point variable	intercept	n follicle CD23	grading follicle CD23	grading intrathymic fat	grading atrophy	
QMG-drop M12-36	<0.001	0,914	0,974	0,904	0,971	logistic regression
MMS M12-36	<0.001	0,402	0,847	0,082	0,621	
QMG-score at baseline	0,006	0,489	0,479	0,575	0,712	linear regression
MG-duration	0,028	0,363	0,992	0,48	0,301	
Absolute prednisone dose before surgery	0,05	0,47	0,386	0,129	0,725	
Absolute prednisone dose after surgery	0,674	0,755	0,553	0.015 *	0,741	

Translating these statistical findings into common clinicopathological terms, it appears that thymectomy specimens with more heterogeneous fat distribution show a trend for higher postoperative prednisone requirements, which is a surrogate for a more delayed clinical improvement. However, due to the great heterogeneity of our data, this correlation is not sufficient to predict clinical outcome.

## Discussion

### 4.1 Advantages and limitations of using entropy as measure of heterogeneity

Generally speaking, entropy belongs to the few basic measurable entities in nature. It can be mathematically formulated using different axioms. For example, Shannon’s entropy definition follows a basic additive algorithm (H1+H2 = H3), in contrast to Pincus’s entropy which includes a non-zero additional term dependent upon boundary conditions [23]. Having spatial data, as shown above, Shannon’s entropy could easily be calculated for all obtained parameters. In this context we could show that it is an independent and valuable new variable: The entropy of a given parameter showed other correlations than the parameter itself. For instance, the entropy of the intra-thymic fat content was independent of age (compare section 8.2), whereas intrathymic fat content showed a correlation with age (because of thymic involution). Vice versa, the entropy of intrathymic fat content showed a correlation to the BMI, whereas the parameter itself showed no correlation.

**Fig 4:**
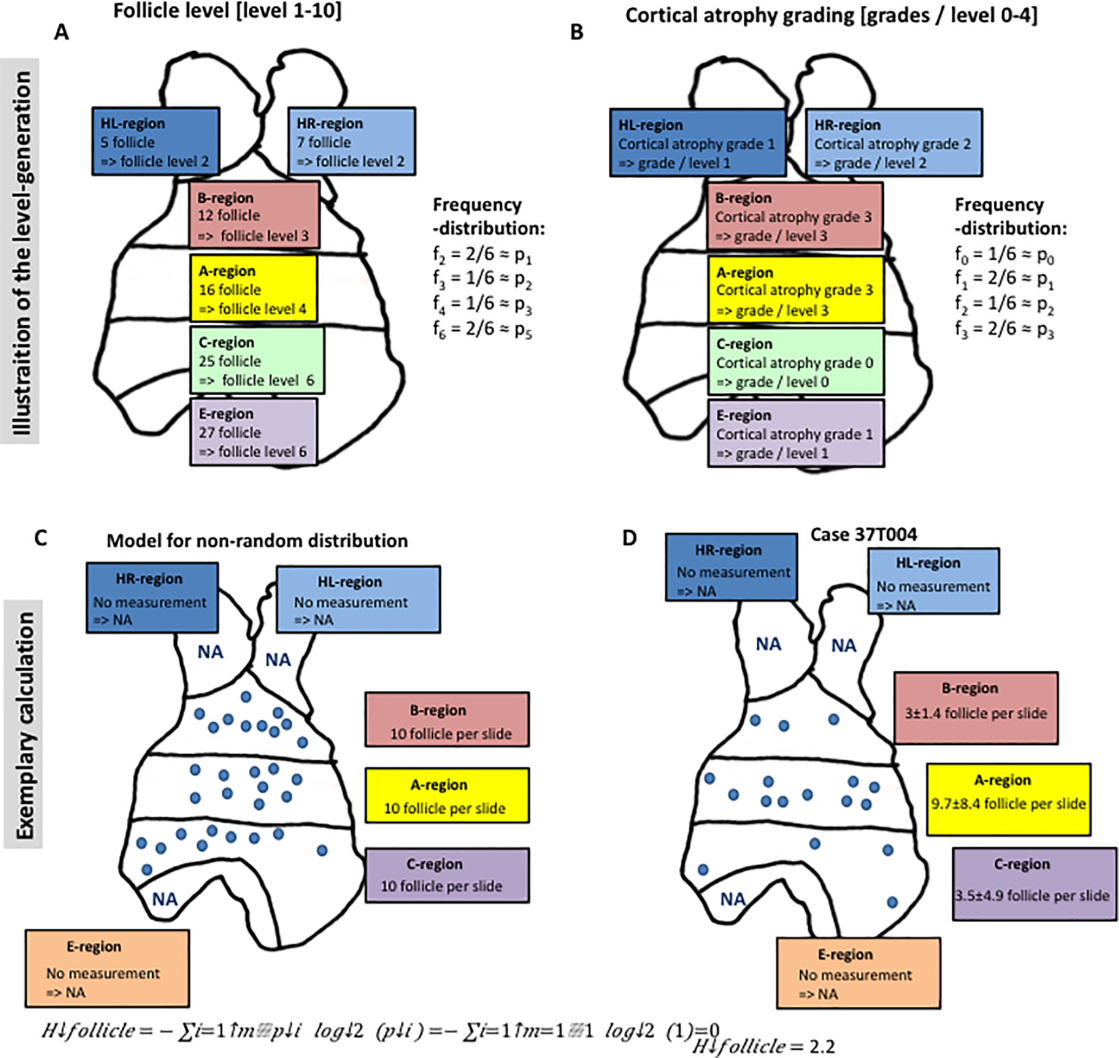
Scheme to illustrate the definition of levels and the calculation of entropy that is based thereon. A-B): For one fictional case the spatial heterogeneity and the in-case variance for one variable is illustrated (number of follicles per slide in Fig 2A and atrophy grading in Fig 2B). The numbers of follicles per slides were mapped to one of 10 levels (with an interval of 5 lymphoid follicles; accordingly level 0 = <5 follicles; level 1 = 5 - < 10; etc.). The resulting frequencies / relative probabilities are plotted next to the scheme. C-D): Entropy as calculated for the number of follicles in case 37T004 C) Fictional thymus with a non-random, completely homogenous distribution of measurements for the number of follicles. The corresponding entropy equals zero. D) Mean-values and standard deviations of the number of follicles per region in CD23-stained sections of case 37T004. The entropy value in this case with heterogeneously distributed follicle frequencies across the various regions is 2.2bit.

Basically, the relation of the entropy to the number of measurement points is one possible limitation. However, we could rule out this potential flaw as illustrated in section 8.3: We did not find a significant correlation between the sample sizes and entropy values using our data. However, regarding the strategy of tissue work-up, the strong relation between entropy and the number of sample points argues for an exentsive work-up scheme with sufficiently spaced sampling point: Too few or too closely located sampling points would reduce the meaningfulness of the resulting entropy value. Another possible limitation is the choice of the number of parameter levels (compare Fig 4A and 4B): For interval scaled variables (e.g. the number of follicles in Fig 4A), an appropriate number of chosen levels will result in entropy values that are robust against minimal variations, thereby avoiding over-estimation of disorder / heterogeneity. On the other hand, if the number of chosen levels is too low, entropy will appear deceptively low, pretending a high degree of order / homogeneity. Accordingly, we deemed 10 levels with an interval of 5 follicles as reasonable compromise that is similar to the choice of levels that is commonly used in image processing software [12].

### 4.2 New perspectives through the introduction of entropy in digital pathology

In imaging based disciplines the term “entropy” has mostly been used in the context of image processing as a textural feature. For instance, in radiology the ‘textural entropy’ has been used to predict different stages of rectal cancer [24] or the survival of breast cancer patients [25], while, in pathology, it has been used to quantify nuclear features after toxic interventions [26, 27].

However, since it is a tool to measure any type of system complexity, entropy analysis and its variations (e.g. “minimum spanning tree (MST) entropy”) might be useful wherever heterogeneity plays a diagnostic or biological role, be it in cancer or beyond [23, 28–30]: Examples comprise the quantification of heterogeneity of inflammatory infiltrates (e.g. in the fields of tumor immunology, pulmonary interstitial diseases and transplant rejection) or degenerative and regenerative processes (e.g. bone marrow alterations after toxic insults or following hematopoietic stem cell transplantation). For any of these applications no special statistical software would be needed, since entropy can easily be calculated and the features it relies on can be quite heterogeneous in regard to their means of measurement (compare Table 1). In practical terms and in order to determine entropy e.g. of a simple parameter like follicular grade, a pathologist would just need a specimen worked up in more than one section, a microscope (to estimate the parameter on visual inspection) and a spreadsheet (to collect the data). A sophisticated spatial work up scheme like the one used in this work (compare Fig 1) would allow to compare different regions of a specimen (e.g. entropy on the right as compared to the left lobule), but is not mandatory to determine entropy overall.

Beyond the analysis of heterogeneity of tissue images, entropy analysis may help to quantify the heterogeneity of RNA-seq data [31] and the heterogeneity of tumors in terms of subclone composition.[32] Finally, through correlation with clinical endpoints, entropy analysis might have the potential to detect new, quantitative types of predictive biomarkers on the morphological and molecular level in neoplastic as well as reactively changed tissue.

## Appendix A

### MGTX executive committee

Data Coordinating Center: Gary R. Cutter, Inmaculada B. Aban, Greg Minisman, Michelle Feese, Hui-Chien Kuo (Dept. of Biostatistics, University of Alabama at Birmingham, Birmingham, AL).

Trial Leadership: John Newsom-Davis (Department of Neurology, Oxford University, Oxford, U.K.); Gil I. Wolfe (Department of Neurology, University at Buffalo Jacobs School of Medicine and Biomedical Sciences); Henry J. Kaminski (Department of Neurology, George Washington University School of Medicine and Health Sciences); Alfred Jaretzki III, Joshua R. Sonett (Section of General Thoracic Surgery, Columbia University Medical Center, New York, NY).

### MGTX investigators

Argentina: Claudio Mazia, Valeria Saluto, Moises Rosenberg, Valeria Alvarez, Lisa Rey (University of Buenos Aires, Buenos Aires).

Australia: John King, Helmut Butzkueven, John Goldblatt, John Carey (University of Melbourne, Melbourne); John Pollard, Stephen Reddel, Nicholas Handel, Brian McCaughan, Linda Pallot (University of Sydney, Sydney).

Brazil: Márcia Waddington-Cruz, Ricardo Novis, Carlos Boasquevisque (Federal University of Rio De Janeiro, Rio De Janeiro); Elza Dias-Tosta, Rubens Morato-Fernandez, Manoel Ximenes (Hospital de Base do Distrito Federal, Brasília); Lineu Werneck, Rosana Scola, Paulo Soltoski (Universidade Federal do Parana; Curitiba).

Canada: Colin Chalk, Fraser Moore, David Mulder, Lisa Wadup (McGill University, Montreal, QC); Joel Oger, Michele Mezei, Kenneth Evans, Theresa Jiwa, Anne Schaffar (University of British Columbia, Vancouver, BC); Chris White, Cory Toth, Gary Gelfand, Susan Wood (University of Calgary, Calgary, AB); Elizabeth Pringle, Jocelyn Zwicker, Donna Maziak, Farid Shamji, Sudhir Sundaresan, Andrew Seely (University of Ottawa, Ottawa, ON).

Chile: Gabriel Cea, Renato Verdugo, Alberto Aguayo (University of Chile, Santiago).

Germany: Sebastian Jander, Philipp Zickler, Michael Klein (University of Düsseldorf, Düsseldorf); Alexander Marx, Philipp Ströbel, Cleo-Aron Weis (University of Heidelberg, Mannheim); Arthur Melms, Felix Bischof, Hermann Aebert, Gerhard Ziemer (University of Tübingen, Tübingen); Wilfred Nix, Björn Thümler, Thomas Wilhem-Schwenkmezger, Eckhard Mayer (Johannes-Gutenberg University, Mainz); Berthold Schalke, Peter Pöschel, Gisela Hieber (University of Regensburg, Regensburg); Karsten Wiebe (University of Münster, Münster).

Italy: Giovanni Antonini, Alessandro Clemenzi, Vanessa Ceschin, Erino Rendina, Federico Venuta, Stefania Morino, Elisabetta Bucci (University of Rome “Sapienza,” Rome); Luca Durelli, Alessia Tavella, Marinella Clerico, Giulia Contessa, Piero Borasio (University of Torino, Torino); Amelia Evoli, Serenella Servidei, Pierluigi Granone (Catholic University, Rome); Renato Mantegazza, Emilia Berta, Lorenzo Novellino, Luisa Spinelli (National Neurological Institute “Carlo Besta,” Milan).

Japan: Masakatsu Motomura, Hidenori Matsuo, Takeshi Nagayasu (Nagasaki University, Nagasaki); Hiroaki Yoshikawa, Masaharu Takamori, Makoto Oda, Isao Matsumoto, Yutaka Furukawa, Daisuke Noto, Yuko Motozaki, Kazuo Iwasa, Daisuke Yanase (Kanazawa University, Kanazawa).

Mexico: Guillermo Garcia Ramos, Bernardo Cacho, Lorenzo de la Garza (Instituto Nacional de la Nutrición, Tialpan).

Poland: Anna Kostera-Pruszczyk, Marta Lipowska, Hubert Kwiecinski, Anna Potulska-Chromik (Medical University of Warsaw, Warsaw); Tadeusz Orlowski (Institute of Tuberculosis and Lung Disease, Warsaw).

Portugal: Ana Silva, Marta Feijo, António Freitas (Porto University, Porto).

South Africa: Jeannine Heckmann, Andrew Frost, Edward Lee Pan, Lawrence Tucker, Johan Rossouw, Fiona Drummond (University of Cape Town, Cape Town).

Spain: Isabel Illa, Jorge Diaz, Carlos Leon (H. Sant Pau, Universitat Autònoma de Barcelona, Barcelona).

Taiwan: Jiann-Horng Yeh, Hou-Chang Chiu, Yei-San Hsieh (Fu-Jen Catholic University, Taipei).

Thailand: Rawiphan Witoonpanich, Supoch Tunlayadechanont, Sukasom Attanavanich (Ramathibodi Hospital, Mahidol University, Bangkok).

The Netherlands: Jan Verschuuren, Chiara Straathof, Maarten Titulaer, Michel Versteegh, Arda Pels, Yvonne Krum (Leiden University, Leiden).

United Kingdom: Camilla Buckley, M. Isabel Leite, Angela Vincent, David Hilton-Jones, Chandi Ratnatunga, John Newsom-Davis (University of Oxford, Oxford); Maria Elena Farrugia, Richard Petty, James Overell, Alan Kirk (Queen Elizabeth University Hospital, Glasgow); Andrew Gibson, Chris McDermott, David Hopkinson (University of Sheffield, Sheffield); Bryan Lecky, David Watling, Dot Marshall, Sam Saminaden, Deborah Davies, Charlotte Dougan, Siva Sathasivam, Richard Page, Dot Marshall (Walton Centre for Neurology and Neurosurgery, Liverpool Heart and Chest Hospital, Liverpool); Jon Sussman, John Ealing, Peter Krysiak (University of Manchester, Manchester).

United States: Anthony Amato, Mohammad Salajegheh, Michael Jaklitsch, Kristen Roe (Brigham and Women’s Hospital, Boston, MA); Tetsuo Ashizawa, Robert Glenn Smith, Joseph Zwischenberg, Penny Stanton (University of Texas Medical Branch, Galveston, TX); Alexandru Barboi, Safwan Jaradeh, William Tisol, Mario Gasparri, George Haasler, Mary Yellick, Cedric Dennis (Medical College of Wisconsin, Milwaukee, WI); Richard Barohn, Mamatha Pasnoor, Mazen Dimachkie, April McVey, Gary Gronseth, Arthur Dick, Jeffrey Kramer, Melissa Currence, Laura Herbelin (University of Kansas, Kansas City, KS); Jerry Belsh, Geoge Li, John Langenfeld, Mary Ann Mertz (Robert Wood Johnson University, New Brunswick, NJ); Michael Benatar (University of Miami, Miami, FL); Taylor Harrison, Seth Force, Sharon Usher (Emory University, Atlanta, GA); Said Beydoun, Frank Lin, Steve DeMeester, Salem Akhter, Ali Malekniazi, Gina Avenido (University of Southern California, Los Angeles, CA); Brian Crum, Margherita Milone, Stephen Cassivi, Janet Fisher (Mayo Clinic, Rochester, MN); Emma Ciafaloni, Chad Heatwole, Thomas Watson, James Hilbert, Alexis Smirnow (University of Rochester, Rochester, NY); B. Jane Distad, Michael Weiss, Douglas Wood, Joanna Haug (University of Washington, Seattle, WA); Raina Ernstoff, Jingyang Cao, Gary Chmielewski, Robert Welsh, Robin Duris (William Beaumont Hospital, Royal Oak, MI); Laurie Gutmann, Gauri Pawar, Geoffrey Marc Graeber, Patricia Altemus, Christopher Nance, Ludwig Gutmann (West Virginia University, Morgantown, WV); Carlayne Jackson, Patrick Grogan, John Calhoon, Pamela Kittrell, Deborah Myers (University of Texas Health Science Center, San Antonio, TX); Henry Kaminski, Ghazala Hayat, Keith Naunheim, Susan Eller, Eve Holzemer (St. Louis University, St. Louis, MO); Bashar Katirji, Amer Alshekhlee, Jason Robke, Brenda Karlinchak (Case Western Reserve University, Cleveland, OH); Jonathan Katz, Robert Miller, Ralph Roan, Dallas Forshew (California Pacific Medical Center, San Francisco, CA); John Kissel, Bakri Elsheikh, Patrick Ross, Sharon Chelnick (The Ohio State University Wexmer Medical Center, Columbus, OH); Richard Lewis, Agnes Acsadi, Frank Baciewicz, Stacey Masse (Wayne State University, Detroit, MI); Janice Massey, Vern Juel, Mark Onaitis, James Lowe, Bernadette Lipscomb (Duke University, Durham, NC); Tahseen Mozaffar, Gaby Thai, Jeffrey Milliken, Veronica Martin, Ronnie Karayan (University of California, Irvine, CA); Suraj Muley (Barrow Neurological Institute, Phoenix, AZ); Gareth Parry, Sara Shumway (University of Minnesota, Minneapolis, MN); Shin Oh, Gwen Claussen, Liang Lu, Robert Cerfolio, Angela Young, Marla Morgan (University of Alabama at Birmingham, Birmingham, AL); Robert Pascuzzi, John Kincaid, Kenneth Kesler, Sandy Guingrich, Angi Michaels (Indiana University, Indianapolis, IN); Lawrence Phillips, Ted Burns, David Jones, Cindy Fischer (University of Virginia, Charlottesville, VA); Michael Pulley, Alan Berger, Harry D’Agostino, Lisa Smith (University of Florida, Jacksonville, FL); Michael Rivner, Jerry Pruitt, Kevin Landolfo, Demetric Hillman (Augusta University, Augusta, GA); Aziz Shaibani, Angelo Sermas, Ross Ruel, Farah Ismail (Nerve and Muscle Center of Texas, Houston, TX); Mark Sivak, Martin Goldstein, Jorge Camunas, Joan Bratton (Mount Sinai Hospital, New York, NY); Rup Tandan, Hill Panitch, Bruce Leavitt, Marilee Jones (University of Vermont, Burlington, VT); Gil Wolfe, Srikanth Muppidi, Steven Vernino, Sharon Nations, Dan Meyer, Nina Gorham (University of Texas Southwestern, Dallas TX).
